# Evaluation of EDTA and silver nanoparticle based root canal irrigants on smear layer elimination and dentine surface roughness

**DOI:** 10.6026/973206300220118

**Published:** 2026-01-31

**Authors:** Vathsala N, Abhishek Mehta, Vaibhav Tammanna Chougule, Nilabh Kalita, Sourav Chandra Bidyasagar Bal, Radhika V, Shail D. Kushwaha, Vardharajula Venkat Ramaiah

**Affiliations:** 1 Department of Conservative Dentistry and Endodontics, The Oxford Dental College and Hospital, Bangalore, Karnataka, India; 2 Department of Conservative Dentistry and Endodontic, Daswani Dental College and Research Centre, Kota, Rajasthan, India; 3 Department of Pedodontics, Bharati Vidyapeeth (Deemed to be University) Dental College and Hospital, Sangli, Maharashtra, India; 4 Intern, Government Dental College, Dibrugarh, Assam, India; 5 Department of Public Health Dentistry, Institute of Dental Sciences, S "O" A Deemed to be University, Bhubaneswar, Odisha, India; 6 Department of Conservative Dentistry and Endodontics, Jaggi Dental Care, Trichy, India; 7 Intern, Kalinga Institute of Dental Sciences, KIIT Deemed to be University, Patia, Bhubaneswar-751024, Odisha, India; 8 Department of Public Health, College of Medical Sciences, Qassim University, Buraydah, Saudi Arabia

**Keywords:** Ethylene diaminetetraacetic acid (EDTA), root canal irrigants, smears layer, silver nanoparticle, surface roughness

## Abstract

Successful endodontics procedure is based on complete biomechanical preparation and three dimensional obturaion. Root canal irrigation is to eliminate necrotic pulp tissue remains and smear layers. There are limited reported studies on comparison of nanoparticle based irrigants. Therefore, it is of interest to assess the efficacy of nanoparticle, EDTA irrigants and sodium hypochlorite on smear layer elimination and surface roughness on root canal dentine. Sodium hypochlorite followed by Silver nanoparticle based irrigant showed good efficacy in removal of smear layer. Silver nanoparticle showed minimal surface roughness of root canal dentine compared to sodium hypochlorite and EDTA.

## Background:

Complete biomechanical preparation of root canals and three-dimensional obturation are essential for successful endodontic procedures [1]. Root canal irrigation serves the purposes of removing smear layers, necrotic tissue remnants, and microbes and their by-products [2, 3-4]. The success rate in root canals is determined by an irrigant's capacity to break up the biofilm and eradicate the bacterial load. Effective outcomes cannot be achieved by mechanical cleaning and shaping alone due to the complicated anatomy of root canals and the polymicrobial load. On the other hand, adding a possible irrigant guarantees that the bacterial load is removed as much as possible while avoiding reinfections. The most popular root canal irrigants are EDTA, hydrogen peroxide, sodium hypochlorite, and chlorhexidine [5]. No single solution has yet to fully satisfy all the parameters of the ideal endodontic irrigant, despite the fact that numerous irrigants have been offered to improve root canal disinfection [4]. Nowadays, sodium hypochlorite (NaOCl) is regarded as the gold standard [6]. In clinical settings, sodium hypochlorite (NaOCl) with a concentration of 2.5-5% has been a typical irrigation solution. This irrigation solution has antimicrobial qualities and can disintegrate organic tissues [2]. Nevertheless, it has a number of disadvantages, such as an unpleasant taste, toxicity, partial smear layer removal, and detrimental effects on dentin flexural strength [7]. Certain root canal irrigants like NaOCl and ethylene diaminetetraacetic acid (EDTA), can alter dentin's structural properties, like as microhardness, which in turn affects the coronal restoration and obturation quality [8]. As a result, several efficient root canal irrigants were tested, including those based on herbs and nanoparticles.

Because it can react with calcium ions in dentin to generate calcium chelation, which dissolves the inorganic tissues from smear layers, ethylenediaminetetraacetic acid (EDTA) is frequently used as final irrigant. Long-term EDTA exposure, however, may change the dentin's structural properties, compromising its mechanical integrity and causing erosion [9]. Attempts have recently been undertaken to create novel and enhanced irrigants with the highest level of biocompatibility and antibacterial qualities. Nanoparticle inclusion is one such approach [5]. A remarkable development over the past ten years, nanotechnology has completely changed scientific research and development. Certain nanoparticles, including silver (AgNPs), have exceptional antibacterial qualities [10]. Other metal nanoparticle with promising antibacterial effect, even against microbes resistant to antibiotics, and positive characteristics for use in endodontics is zinc oxide (ZnONPs) [11]. Because of its ability to break bacterial cell membranes and interact electrostatically, AgNp has long been utilized as an antibacterial agent. AgNP's small size and decreased surface area facilitate easy penetration and improved antibacterial qualities when compared to conventional metal particles [12]. For centuries, silver has been utilized as a non-toxic, safe inorganic antibacterial agent that can eradicate over 650 different kinds of germs [13]. There are limited reported studies on comparison of nanoparticle based irrigants. Therefore, it is of interest to assess the efficacy of nanoparticle, EDTA and sodium hypochlorite irrigants on smear layer removal and surface roughness of root canal dentine.

## Materials and Methods:

Intact, healthy 36 straight, single-rooted mandibular premolars without any pathology that were extracted for orthodontic purposes were used in this *in vitro* study. All teeth were cleaned and sterilized. The working lengths were then subtracted by 1 mm from the root lengths after all teeth were seperated at the cementoenamel junction, leaving a root length of 12 mm. In order to replicate the clinical situation, the sticky wax was then used to seal the root apices. Using rotary endodontic files, an aseptic crown- down approach was used for access opening, pulp extraction, and root canal preparation. Then total 36 teeth were randomly divided into 3 groups with equal sample size of 12 each based on type of irrigants used as; Group I: silver nano particle based, Group II: 17% EDTA (Pulpdent, Watertown, MA, USA) based irrigants and Group III: sodium hypochlorite based irrigant.

Commercially available EDTA and Sodium hypochlorite solutions were used for the study. Silver nitrate (AgNO3) was added to the cell- free supernatant that was obtained from centrifuging the incubated broth of Bacillus in order to synthesize AgNps from the probiotic of the Bacillus mixture. The mixture was then incubated in a shaker incubator and centrifuged; the supernatant was discarded, and the deposit at the bottom of the tube represented the collection of the nanoparticles. To prevent AgNO3 oxidation, every step was completed in the dark. Ultraviolet (UV)-visible spectroscopy was used to characterize the Ag nanoparticles. Cleaning and shaping of the root canals was done along with irrigation using respective irrigants for 5 min. Paper points were then used to dry the canals. Every root was sectioned into mesial and distal pieces after being vertically grooved in a bucco-lingual orientation. Half of the root was used for surface roughness assessment, and the other half was used for smear layer evolution. A digital roughness tester (SR 300, Taylor Hobson, Leicester, England) was used to measure each sample's surface roughness. The Ra parameter, which can be defined as the numerical average value of the roughness profile from the center line inside the measuring length, determines the overall roughness of the surface. A scale of 1 to 4 was used to score the degree of smear layer removal. Score 1: All tubules have been cleaned and opened, and there is absolutely no smear layer or debris. Score 2: The majority of tubules have been cleansed and opened, with a few spots covered with debris and a smear layer. Score 3: Few tubules are open, and nearly all surfaces are covered in a smear layer and debris. Score 4: Every surface is covered in garbage and a smear layer. An SEM (JEOL JSM-5510, Tokyo, Japan) was used to analyze the canal wall of the apical third of each root. Data obtained were analyzed used IBM statistical softwere version 24.0 using two-way ANOVA, followed by the Tukey test and Mann-Whitney U with a significance level of 0.05.

## Results:

Table 1 indicated that, sodium hypochlorite had highest smear layer removal followed by silver nanoparticle irrigants and least with EDTA (P<0.005). Table 2 indicated that silver nanoparticle irrigants had lowest surface roughness followed sodium hypochlorite by and heighest with EDTA (P<0.001).

## Discussion:

Eliminating bacteria from the root canal system is the primary goal of endodontic treatment with the intention of long-term success. Access opening, working length determination, cleaning and shaping, and canal obturation are the four stages of root canal therapy. Cleaning, shaping, and irrigation are crucial steps in the root canal therapy process. Root canal irrigants should be biocompatible, antimicrobial, nontoxic, and effective against both aerobic and anaerobic organisms. They should also have the ability to lubricate and break up biofilm [5]. The complex structure of the root canal system allows microorganisms to persist even after routine cleaning is finished. *Enterococcus faecalis* is a facultative anaerobic microbe that typically lives in the oral flora [14]. Because of their superior antibacterial qualities, silver nanoparticles (AgNPs) are used in disinfection because they are efficient against a variety of pathogens, including *E. faecalis* [15]. In an attempt to enhance root canal disinfection, nanoparticles have also been investigated in the endodontic area [16]. In the current investigation, irrigants based on silver nanoparticles demonstrated good efficacy in eliminating the smear layer on root canal dentine with the least amount of surface roughness. One possible explanation for EDTA's potent chelation effect is its increased capacity to demineralize the smear layer, particularly its inorganic components. Erosion may result from this demineralization impact, particularly in peritubular and intertubular dentin [17]. It can be explained by the fact that NaOCl exclusively dissolves organic molecules and causes the denaturation and disintegration of dentin collagen [2].

Irrigants containing ZnONPs and AgNPs considerably increased the root dentin microhardness, according to Sahebi *et al.* [18]. Ratih *et al.* investigated the effects of chitosan nanoparticles as a final irrigation solution on the smear layer elimination, micro-hardness, and surface roughness of root canal dentin. They came to the conclusion that, in comparison to 17% EDTA, final irrigation with 0.2% chitosan nanoparticles had the same effect on smear layer elimination [2]. In maxillary anterior teeth with necrotic pulp, Sevagaperumal *et al.* assessed the antimicrobial efficiency of graphene oxide (GO) silver nanoparticle (AgNp) as root canal irrigant (RCI) with other RCIs. They found that GO AgNp irrigant is an effective biocompatible antimicrobial agent that is superior to 2% chlorhexidine and normal saline and comparable to 2.5% sodium hypochlorite [5]. When silver nanoparticles were utilized as an irrigation solution, AL-Fhham found that they were just as effective against *E. faecalis* biofilm as sodium hypochlorite [14]. Before applying the sealer, Razumova *et al.* found that a nano-silver solution with a particle size of 1-2 nm demonstrated its capacity to enter the dentinal surfaces and form a final layer covering the dentinal surface of the root canal [13]. When combined with silver nanoparticles, sodium hypochlorite had more antibacterial activity than the other studied groups, according to Chhabra *et al.* [19]. In relationship to sodium hypochlorite and chlorhexidine solutions, Rodrigues *et al.* assessed the antibacterial efficacy of an irrigant comprising silver nanoparticles in an aqueous carrier against *E. faecalis* biofilm and infected dentinal tubules. In comparison to solutions frequently used in root canal therapy, the researchers found that the AgNP irrigant was less effective against *E. faecalis* [20]. In addition to eliminating the smear layer and organic and inorganic waste from the root canal dentine, irrigating treatments help disinfect canals [21]. Endodontic therapy uses chemical washing to sanitise the complex root canal system [22]. In the present study we found good efficacy in removal smear layer by AgNP based irrigant. Use of AgNP helps improving the success root canal therapy because of its broad-spectrum antibacterial action, Enhanced Penetration, advantageous for root canal disinfection and improved canal cleaning efficacy. To validate the results, more clinical research with a bigger sample size is needed.

## Conclusion:

Sodium hypochlorite followed by Silver nanoparticle based irrigant showed good efficacy in removal smear layer. Silver nanoparticle showed minimal surface roughness compared to sodium hypochlorite and EDTA. It can be concluded that, silver nanoparticles can be used as an adjunct to sodium hypochlorite solution.

## Figures and Tables

**Table 1:** Impact of irrigation treatments on root canal dentin smears layer removal

**Irrigant type used**	**Mean Smear Layer Removal scoring**	**p**
Group I- Silver nano particle irrigant	2	0.005
Group II- EDTA	1	
Group III: Sodium hypochlorite	3	

**Table 2:** Impact of irrigation solutions on root canal dentin surface roughness

**Irrigant type used**	**Mean Surface roughness scoring (µm)**	p
Group I- Silver particle irrigant	0.65 ± 0.42	0.001*
Group II- EDTA	2.56 ± 0.07	
Group III: Sodium hypochlorite	0.76 ± 0.31	
*significant
